# Socioeconomic Determinants of Career Intention in Pharmacy Students in Vietnam

**DOI:** 10.3390/pharmacy13060161

**Published:** 2025-11-02

**Authors:** Quang Ngoc Phan, Oanh Thi Kim Nguyen, Hoa Thi Tran, Ngoc Bao Dang, Nam Hoang Tran

**Affiliations:** 1 The Center Service for Technology Science of Medi-Phar., Thai Binh University of Medicine and Pharmacy, Hung Yen 410000, Vietnam; 2 Faculty of Pharmacy, Thai Binh University of Medicine and Pharmacy, Hung Yen 410000, Vietnam; 3 Global Citizenship Foundation, 139, Sector 14, Sonipat Haryana 131001, India; 4 Research Center for Higher Education, Tokushima University, Tokushima 770-8502, Japan

**Keywords:** career intention, family encouragement, gender differences, healthcare workforce, pharmacy students, socioeconomic determinants, urban–rural disparities, Vietnam

## Abstract

Background: The pharmacy workforce in Vietnam is rapidly evolving, but little is known about how gender and socioeconomic factors shape career intentions and sector preferences of students. Understanding these determinants is essential for healthcare workforce planning. Objective: To investigate how gender and socioeconomic determinants influence intention of pharmacy students to pursue a career and their preferred sector in Vietnam. Methods: A cross-sectional survey was conducted among 462 students from 2nd to 5th year at a Vietnamese university. Demographic data, socioeconomic background, and career intentions were analyzed using chi-squared tests, multinomial logistic regression, and binary logistic regression. Results: Of 462 respondents, 71.9% intended to pursue a pharmacy career, 2.6% reported no, while 25.5% were unsure. Gender differences were evident but did not reach statistical significance (*p* = 0.083). Female students were more likely to choose clinical, hospital pharmacy or regulation, whereas male students showed higher preference for community, industry and academia. Binary logistic regression revealed that urban origin (OR = 1.34, 95% CI = 1.01–1.78, *p* = 0.041) and family encouragement (OR = 2.53, 95% CI = 1.60–3.99, *p* < 0.001) significantly predicted career intention, while gender and income were non-significant. Conclusions: Family encouragement and urban upbringing influence pharmacy career pursuit, while gender may shape sectoral preferences. Policies should address gender equity and enhance support for students from rural or disadvantaged backgrounds.

## 1. Introduction

Healthcare workforce development has increasingly become a global policy priority, with pharmacy playing a central role in healthcare delivery. International studies have shown that gender and socioeconomic determinants significantly shape career trajectories in pharmacy and related health professions. For instance, in high-income countries such as the United States and the United Kingdom, female pharmacy graduates are more likely to pursue community and clinical roles, whereas male graduates tend to enter managerial or industry-related pathways [1,2,3]. Similar patterns have been documented across Europe and North America, where gender continues to influence not only career entry but also progression and leadership opportunities in pharmacy [4,5,6,7]. In emerging economies, including India and Jordan, socioeconomic background and family expectations strongly influence whether students pursue pharmacy careers at all, reflecting the continued salience of cultural and financial factors in shaping professional aspirations [8,9,10,11,12,13]. Comparative studies from Southeast Asia further indicate that students from rural and lower-income families often face barriers to entering specialized sectors of pharmacy, underscoring inequities that workforce policies must address [12,13,14,15]. Taken together, this body of evidence highlights the intersection of gender roles, family influence [16,17], and socioeconomic constraints as critical determinants of pharmacy career intentions worldwide.

In Vietnam, pharmacy education has expanded rapidly in recent decades in response to rising demand for healthcare professionals [4,18,19]. The profession, however, faces persistent structural and social challenges. Most pharmacy students originate from rural areas, where access to higher education and exposure to diverse career pathways are limited, creating disparities in professional orientation [18,19]. Gender disparities also remain salient, with women overrepresented in the student population but underrepresented in leadership, industrial, and regulatory roles [20,21]. Moreover, socioeconomic inequalities, including household income, parental education, and scholarship opportunities, shape both access to training and career aspirations, often constraining students from lower-income backgrounds to prioritize stability and immediate employment over advanced specialization [22,23]. Family expectations further reinforce these dynamics: Vietnamese families continue to exert strong influence on their children’s professional decisions, particularly in medicine and pharmacy, where social prestige and financial stability are perceived as critical [17,24]. Despite the growing importance of pharmacy in national health outcomes, empirical evidence on how gender and socioeconomic factors interact to influence sector-specific career intentions among pharmacy students in Vietnam remains scarce. Addressing this gap can provide critical insights for workforce planning, educational policy, and gender equity in healthcare professions.

Pharmacy sector in Vietnam expands rapidly over the past two decades, reflecting both rising healthcare demand and increased private sector. As of 2024, the country hosts more than 70,000 licensed community pharmacies and over 250 pharmaceutical manufacturers, including foreign-invested companies that play major roles in production and distribution [18]. The sector remains dominated by retail and distribution, while hospital and clinical pharmacy continue to develop as specialized practice areas. Pharmacy education in Vietnam follows a five-year undergraduate model leading to the Bachelor of Pharmacy degree, jointly regulated by the Ministry of Education and Training and the Ministry of Health. As of 2020, over 30,000 students were enrolled in undergraduate pharmacy programs nationwide, including over 12,000 in public universities and over 18,000 in private institutions [4,23]. More than 20 public and private universities now offer accredited pharmacy programs, but variation in curriculum quality and clinical exposure persists across institutions, but pharmacy programs are unevenly distributed across regions, with the majority concentrated in major urban centers such as Hanoi and Ho Chi Minh City [4,19]. This evolving structure underscores both the opportunities and challenges in aligning pharmacy education with the rapidly diversifying pharmaceutical and healthcare markets in the country.

Literature gaps also persist regarding the interaction between gender and family encouragement in shaping career pathways. While gender disparities in pharmacy and health professions have been widely documented, less is known about how family encouragement interacts with these disparities to influence career trajectories. Family expectations and social prestige remain highly influential in many Asian societies, where parental involvement often extends beyond education into shaping professional choices [16,24]. In contexts such as India and Jordan, family encouragement has been found to moderate the effect of gender, with male students more responsive to parental approval in choosing pharmacy or medicine, while female students often experience subtle constraints to pursue roles deemed socially appropriate [9,10,12]. Studies from China and Malaysia similarly highlight the interplay between cultural gender norms and family expectations, suggesting that encouragement can act as both an enabler and a barrier depending on societal context [11,13,14].

In Vietnam, although family influence in education is strong, its gender-specific dynamics within pharmacy remain underexplored. Existing research on Vietnamese higher education has shown that family encouragement strongly predicts students’ persistence and career motivation [4,17,23], but these studies have not disaggregated the effects by gender. Given that pharmacy is both female-dominated in enrollment and gender-stratified in professional outcomes, the absence of systematic study on the moderating role of gender is a critical gap. Addressing this gap can provide insights into how structural factors (socioeconomic background, rural–urban disparities) and social dynamics (gender norms, parental influence) jointly shape pharmacy students’ career decisions, with important implications for designing policies that promote gender equity and optimize workforce distribution in Vietnam [18,25].

Against this background, the present study investigates the following research questions: (1) How do gender and socioeconomic background shape pharmacy students’ intention to pursue a pharmacy career? (2) How do these factors influence the choice of career sector (e.g., community, hospital, clinical, industry, academia, or regulation)? (3) Does gender moderate the influence of family encouragement and other social factors on career intention? Based on prior international and regional literature, we hypothesize that female students will be more likely to prefer patient-facing sectors, while male students will be more inclined toward industry and regulation; and that family encouragement will strongly predict career intention, with potentially greater influence among male students.

## 2. Materials and Methods

### 2.1. Study Design and Participants

This study employed a cross-sectional survey design conducted in September 2025 among undergraduate pharmacy students at a large public university in Vietnam. Eligible participants included students in the second to fifth years of study; first-year students were excluded because they had not yet commenced core pharmacy coursework. Although formal career orientation sessions are not part of the curriculum at the study institution, second- and third-year students were included to capture early perceptions and developing awareness of pharmacy career intentions prior to the professional training years. The questionnaire was administered in an online format. To ensure data integrity and prevent multiple submissions, the ‘Hide Submit another response’ function in Microsoft Forms was enabled, and the dataset was manually screened for duplicate demographic entries before analysis. A total of 550 students were approached, of whom 462 completed questionnaires, resulting in a response rate of 84%, representing a broad distribution across academic years. The minimum sample size was calculated using Cochran’s formula (*n* = Z^2^pq/e^2^) for categorical data with a 95% confidence level, 5% margin of error, and an assumed response proportion (*p*) of 0.5, resulting in a required sample of 384. To ensure sufficient statistical power and allow for potential non-response, all 550 students enrolled from the 2nd to 5th years, the entire eligible student population of the Faculty of Pharmacy, were invited. The sample size was considered adequate for multivariate regression analyses and provided sufficient power to detect meaningful associations between gender, socioeconomic background, and career intentions.

### 2.2. Instrument

Data were collected using a structured questionnaire initially developed in English and subsequently translated into Vietnamese through a forward–backward translation process to ensure accuracy and cultural relevance. The instrument was developed by the authors, partially adapted from international studies on pharmacy students’ career intentions and related factors [9,11,12,13]. Content validity was established through expert review by three faculty members in pharmacy education and social sciences, and a pilot test with 20 students was conducted to refine clarity and structure. The reliability of attitudinal and perception items was confirmed for internal consistency (Cronbach’s α = 0.81). The final questionnaire consisted of seven sections:

1. Demographics—Age, gender, year of study, and marital status.
2. Socioeconomic background—Household income, parental education, urban–rural origin, scholarship status, and whether respondents had relatives working in pharmacy.
3. Academic self-assessment—Self-rated abilities in biology, chemistry, mathematics, and physics (five-point Likert scale), as well as perceived preparedness for science-based careers.
4. Career intentions—Intention to pursue a pharmacy career (Yes/No/Not sure), preferred sector (community, hospital, clinical, industry, academia, regulation, or other), and willingness to work in rural areas.
5. Gender perceptions—Attitudes toward gender and pharmacy, including agreement with statements such as “Pharmacy is a female-dominated profession” and “My gender influences my career choice in healthcare” (five-point Likert scale).
6. Lifestyle—Information on part-time employment, study–life balance, and extracurricular activities.
7. Mental health—Screening questions adapted from validated instruments such as the WHO-5 Well-Being Index [26] to capture levels of psychological well-being and potential stress.

To maximize participation, the survey was administered in bilingual format (English and Vietnamese) and delivered online via Microsoft Forms.

### 2.3. Variables

*Independent variables*. Gender was measured as male or female, based on self-report in Section 1 of the questionnaire. Household income was reported in three categories aligned with national benchmarks from the General Statistics Office of Vietnam for lower, middle and higher income, and commonly used in socioeconomic surveys and prior research: under 15 million VND/month, 15–30 million VND/month, and over 30 million VND/month [23]. Parental education was collected separately for mother and father with four categories: below lower secondary, high school graduate, bachelor’s degree, and postgraduate. Urban–rural origin was assessed by asking where the student grew up, with options of urban, suburban, or rural. Scholarship status was recorded as a binary variable (Yes/No) regarding whether the student was currently receiving financial aid. Presence of relatives in pharmacy was measured as a Yes/No item regarding whether students had family members working in pharmacy.

*Mediating and moderating variables*. Family encouragement was assessed by asking whether anyone in the family had encouraged the student to study pharmacy (Yes/No). Gender perceptions and career barriers were measured through Likert-scale items in Section 5 of the questionnaire, including agreement with statements such as “I believe pharmacy is a female-dominated field” and “My gender influences my career choice in the healthcare sector” (rated on a 5-point scale from Strongly Disagree to Strongly Agree). Academic self-assessment was collected in Section 3 by asking students to rate their ability in biology, chemistry, mathematics, and physics on a five-point scale from Very Poor to Excellent, as well as their confidence in succeeding in science-based careers.

*Dependent variables*. Career intention was measured in Section 4 with the question, “Do you intend to pursue a career as a pharmacist?” with response options Yes, No, or Not sure. Students who answered “Yes” were further asked to specify their preferred pharmacy sector, with options of community pharmacy, clinical pharmacy, hospital pharmacy, pharmaceutical industry, academia, government regulation, or other (with an open-text field to specify alternatives such as cosmetics or retail).

### 2.4. Statistical Analysis

All analyses were conducted using IBM SPSS Statistics version 28. Descriptive statistics summarized demographic and socioeconomic characteristics of the sample. Bivariate associations were examined using chi-squared tests for categorical variables and *t*-tests or one-way ANOVA for continuous variables. Two multivariate models were then estimated. A binary logistic regression was performed to examine predictors of career intention (Yes versus No/Unsure), reporting odds ratios (ORs) and 95% confidence intervals (CIs). A multinomial logistic regression was applied to assess predictors of career sector choice, with community pharmacy set as the reference category. Both models included gender, household income, parental education, urban origin, scholarship, family encouragement, and relatives in pharmacy as predictors. Interaction terms, particularly gender and family encouragement, were tested to assess moderating effects. Statistical significance was defined as *p* < 0.05.

### 2.5. Ethics

The survey was conducted anonymously, and participation was voluntary. Informed consent was obtained from all respondents before completing the questionnaire. Ethical approval for this study was granted by the Institutional Review Board of Thai Binh University of Medicine and Pharmacy (Approval No. 844/QD-YDTB, dated 29 April 2025).

## 3. Results

### 3.1. Demographic and Socioeconomic Characteristics

Respondents’ mean age was 20.6 years; 72.7% were female, 26.6% male. Most originated from rural areas (76.8%) and low-income households (<15 m VND/month: 70.3%). Only 8.9% received scholarships (Table 1).

### 3.2. Career Intention and Sector Distribution

71.9% intended to pursue pharmacy career, 2.6% did not, and 25.5% were unsure. Among those intending to pursue, the most common sector was industry (39.8%), followed by clinical (19.9%), community (14.8%), hospital (11.7%), regulation (6.6%), and academia (5.1%) (Table 2).

### 3.3. Gender and Career Sector (Bivariate Analysis)

Female students predominated in clinical, hospital and government regulation sectors, while male students were more concentrated in community, industry and academia. The chi-squared test did not reach conventional significance (χ^2^(6, *N* = 332) = 11.18, *p* = 0.083), though sectoral trends were evident. (Table 3).

### 3.4. Career Sector Choice

None of the predictors reached statistical significance in the multinomial logistic regression model (*p* > 0.05). While several odds ratios, such as those associated with father high-school education, were greater than 1 across some sectors, these values only represent non-significant directional tendencies relative to community pharmacy (reference category) and should not be interpreted as meaningful predictors. Similarly, male gender was associated with odds ratios below 1 across all sectors, suggesting a lower but statistically non-significant likelihood of preferring these sectors compared with females (Table 4). Full regression results, including all predictors and reference groups, are provided in Supplementary Table S1.

### 3.5. Career Intention

Urban origin (OR = 1.34, 95% CI = 1.01–1.78, *p* = 0.041) and family encouragement (OR = 2.53, 95% CI = 1.60–3.99, *p* < 0.001) significantly predicted intention to pursue pharmacy. Gender, household income, parental education, scholarship, and relatives in pharmacy were not significant. The model explained 6.3% of variance (Nagelkerke R^2^) (Table 5).

## 4. Discussion

### 4.1. Key Findings

This study examined the gendered and socioeconomic determinants of pharmacy students’ career intentions in Vietnam. The findings highlight three important patterns. First, a majority of students (71.9%) expressed the intention to pursue a career in pharmacy, consistent with prior research reporting strong professional commitment among health science students [27,28]. Second, gender differences were evident in sectoral preferences: female students were more likely to choose clinical, hospital pharmacy, or regulation, whereas male students tended toward industry, community, and academia. Although gendered trends were observed across sectors, none of the variables significantly predicted sectoral choice in the multinomial model, indicating that these patterns were descriptive rather than inferential. These findings are generally consistent with prior research showing gendered sectoral preferences [9,12,29]. However, the current study revealed an interesting deviation, with male students demonstrating greater inclination toward community pharmacy rather than government regulation roles, although these associations did not reach statistical significance in the multinomial model. Third, in line with prior research, family encouragement and urban/rural origin emerged as significant predictors of career intention. For example, a study in China found that family background variables (parental education, whose opinions count in career decisions, household income) strongly influenced pharmacy students’ career intention across sectors [11]. Another study showed that students from urban backgrounds placed greater value on urban job location [30]. It has been reported that rural-origin students were more likely to express intention to work in rural settings [31].

### 4.2. Comparison with Previous Literature

The observed gendered sectoral trends align with international evidence showing that women disproportionately enter patient-facing roles while men dominate industry and managerial positions [8,14,32]. In the United Kingdom, for example, female graduates were more likely to pursue community pharmacy careers, while male graduates opted for industrial or administrative positions [33]. Similarly, studies from the United States and Canada indicate that male pharmacists are overrepresented in technical and entrepreneurial domains [6].

Socioeconomic determinants also mirror findings from Asian contexts. Research from India and Jordan has demonstrated that family influence and socioeconomic background strongly predict health career intentions [12,34]. In Sudan, family encouragement was identified as one of the strongest motivators for pursuing pharmacy education, particularly among students from rural provinces [35]. The significance of family encouragement in our study supports these findings and underscores the collectivist orientation of Vietnamese society, where parental guidance and family prestige are decisive factors in career choice [16,17].

Interestingly, household income and parental education were not significant predictors in the adjusted models, diverging from studies in high-income countries where socioeconomic capital often shapes access to prestigious sectors [36]. This suggests that in Vietnam, non-material factors such as encouragement and exposure may outweigh material resources in shaping intentions.

### 4.3. Policy and Educational Implications

These findings have several implications for workforce planning. Strategies to diversify gender representation across sectors are warranted. Interventions could encourage male students to enter patient-facing sectors while simultaneously supporting female students to advance into industry, academia, and regulation [37,38]. Second, given the strong role of family encouragement, universities and professional associations should design family engagement programs, such as open days or informational sessions, to ensure accurate perceptions of pharmacy careers are conveyed to parents and relatives. Third, rural-origin students may face barriers such as limited exposure to role models, weaker institutional support, or fewer professional networks [19,39]. Policies aimed at targeted mentorship, scholarships, and career guidance could reduce these inequities.

### 4.4. Limitations

Several limitations should be acknowledged. The cross-sectional design prevents causal inference; intentions may not necessarily translate into actual career outcomes. Self-reported data may also be subject to recall bias or social desirability bias, although anonymity was used to mitigate this risk. In addition, smaller subgroup sizes for some career sectors (such as academia, government regulation) limited statistical power, leading to wide confidence intervals and non-significant estimates. Finally, the study was conducted at a single institution, which may restrict generalizability to the broader Vietnamese pharmacy student population.

### 4.5. Future Directions

Future research should adopt longitudinal designs to track whether career intentions translate into actual employment patterns. Comparative studies across multiple institutions and countries in Southeast Asia would also help identify structural versus cultural determinants of pharmacy career choices. Moreover, qualitative studies exploring how family encouragement operates in practice could deepen understanding of the mechanisms underlying this predictor. Finally, interventions aimed at mentoring rural-origin students and balancing gender representation across sectors should be systematically evaluated for effectiveness. Future research should also include direct items assessing aspirations of students for managerial/entrepreneurial roles, e.g., distinguishing between intentions to become a pharmacy entrepreneur, manage a pharmacy, or work as a staff pharmacist. Such granularity would allow a more precise understanding of gendered and socioeconomic patterns in leadership and business orientation of a pharmacy workforce.

## 5. Conclusions

This study provides new understanding about how socioeconomic background can influence career intentions of pharmacy students in Vietnam. Family encouragement and urban origin emerged as significant predictors of career intention, highlighting the role of social support and structural context. These findings imply the following priorities for policy and practice: (1) developing mentorship and family engagement programs to strengthen career motivation, and (2) reducing barriers faced by students of rural origin by providing opportunities for scholarships and professional exposure. Future research should adopt longitudinal and multi-center approaches to validate these findings and evaluate interventions that support disadvantaged students.

## Figures and Tables

**Table 1:** Demographic and Socioeconomic Characteristics of Respondents (*N* = 462).

Variable	*n*	%
Gender		
Female	336	72.7
Male	123	26.6
Uncertain	3	0.6
Year of study		
Grade 2	127	27.5
Grade 3	95	20.6
Grade 4	118	25.5
Grade 5	122	26.4
Household Income		
<15 m	325	70.3
15–30 m	118	25.5
>30 m	19	4.1
Father highest education		
Junior High	203	43.9
High School	158	34.2
Undergraduate	60	13.0
Graduate	41	8.9
Mother highest education		
Junior High	205	44.4
High School	125	27.1
Undergraduate	85	18.4
Graduate	47	10.2
Urban/rural origin		
Urban	83	18.0
Suburban	24	5.2
Rural	355	76.8
Scholarship status		
Yes	41	8.9
No	421	91.1
Part-time job status		
Yes	222	48.1
No	240	51.9
Relatives in Pharmacy		
Yes	120	26.0
No	342	74.0

**Table 2:** Career Intentions and Preferred Sectors (*N* = 462).

Career Intention	n	%
Intention to pursue a pharmacy career		
Yes	332	71.9
No	12	2.6
Unsure	118	25.5
Preferred Sector among Yes (*N* = 332)		
Community Pharmacy	49	14.8
Clinical Pharmacy	66	19.9
Hospital Pharmacy	39	11.7
Pharmaceutical Industry	132	39.8
Academia	17	5.1
Government Regulation	22	6.6
Other	7	2.1

**Table 3:** Bivariate Association Between Gender and Career Sector Choice (*N* = 332).

Career Sector	Female %	Male %	Total %
Community Pharmacy	67.3	32.7	14.8
Clinical Pharmacy	81.8	18.2	19.9
Hospital Pharmacy	84.6	15.4	11.7
Pharmaceutical Industry	68.2	31.8	39.8
Academia	70.6	29.4	5.1
Government Regulation	81.8	18.2	6.6
Other	100.0	0.0	2.1
Total	74.4	25.6	100

Note. χ^2^(6, *N* = 332) = 11.176, *p* = 0.083.

**Table 4:** Multinomial Logistic Regression Predicting Career Sector Choice (Reference: Community Pharmacy (*N* = 332).

Predictor	Clinical Pharmacy OR (95% CI), *p*	Hospital Pharmacy OR (95% CI), *p*	Industry OR (95% CI), *p*	Academia OR (95% CI), *p*	Government Regulation OR (95% CI), *p*
Gender (Male vs. Female)	0.23 (0.03–2.04), *p* = 0.187	0.34 (0.04–3.14), *p* = 0.340	0.18 (0.02–1.49), *p* = 0.111	0.18 (0.02–1.85), *p* = 0.149	0.33 (0.03–3.32), *p* = 0.348
Family encouragement (Yes vs. No)	0.33 (0.07–1.55), *p* = 0.161	0.61 (0.13–2.87), *p* = 0.533	0.79 (0.19–3.34), *p* = 0.753	0.26 (0.04–1.69), *p* = 0.158	0.66 (0.13–3.37), *p* = 0.613
Relatives in pharmacy (Yes vs. No)	1.55 (0.38–6.26), *p* = 0.538	1.11 (0.26–4.65), *p* = 0.888	1.12 (0.29–4.25), *p* = 0.872	1.44 (0.29–7.23), *p* = 0.657	3.69 (0.69–19.79), *p* = 0.128

Note. OR = Odds Ratio; CI = Confidence Interval. None of the predictors reached statistical significance at *p* < 0.05. Full regression results with all variables are presented in Supplementary Table S1.

**Table 5:** Logistic Regression Predicting Intention to Pursue Pharmacy (Yes vs. No).

Predictor	B	S.E.	Wald	df	Sig.	Exp(B)	95% CI Lower	95% CI Upper
Gender (Male = 1)	−0.097	0.240	0.165	1	0.685	0.907	0.567	1.452
Family income	0.011	0.204	0.003	1	0.955	1.011	0.678	1.509
Urban origin	0.294	0.144	4.169	1	0.041	1.341	1.012	1.778
Scholarship	0.084	0.378	0.050	1	0.824	1.088	0.519	2.282
Family encouragement	0.927	0.232	15.925	1	<0.001	2.528	1.603	3.987
Father education	0.029	0.124	0.056	1	0.813	1.030	0.808	1.313
Constant	−0.430	0.694	0.384	1	0.536	0.651		

## Data Availability

The data presented in this study are available on request from the corresponding author due to privacy and ethical restrictions.

## Supplementary Materials

The following supporting information can be downloaded at: https://www.mdpi.com/article/10.3390/pharmacy13060161/s1, Table S1: Multinomial Logistic Regression Predicting Career Sector Choice (Reference = Community Pharmacy).

## Abbreviations

The following abbreviations are used in this manuscript:

CI	Confidence Interval
DV	Dependent Variable
SPSS	Statistical Package for the Social Sciences
VND	Vietnamese Dong
WHO-5	World Health Organization Five Well-Being Index
