# Supplementary material for: Socioeconomic Determinants of Career Intention in Pharmacy Students in Vietnam
Source: Pharmacy (Basel). 2025 Nov 2;13(6):161. doi: 10.3390/pharmacy13060161 (PMC12641655; doi:10.3390/pharmacy13060161)
Supplement: Supplementary file 1 [file pharmacy-13-00161-s001.zip › pharmacy-3927237-supplementary.pdf]

Table S1. Multinomial Logistic Regression Predicting Career Sector Choice (Reference = Community Pharmacy)

Career Sector	Predictor	OR	95% CI	p-value
**Clinical Pharmacy**	Gender (Male vs. Female)	0.23	0.03–2.04	.187
	Household income (Low vs. High)	~0.00	–	.994
	Household income (Middle vs. High)	~0.00	–	.994
	Father's education (Junior High vs. Postgrad)	2.13	0.25–18.43	.491
	Father's education (High school vs. Postgrad)	2.41	0.29–19.99	.415
	Father's education (Bachelor vs. Postgrad)	0.83	0.08–8.46	.877
	Urban origin (vs. Rural)	0.57	0.11–2.99	.503
	Suburban origin (vs. Rural)	0.59	0.06–6.25	.663
	Scholarship (No vs. Yes)	2.43	0.42–14.17	.323
	Family encouragement (No vs. Yes)	0.33	0.07–1.55	.161
	Relatives in pharmacy (No vs. Yes)	1.55	0.38–6.26	.538
**Hospital Pharmacy**	Gender (Male vs. Female)	0.34	0.04–3.14	.340
	Household income (Low vs. High)	~0.00	–	.994
	Household income (Middle vs. High)	~0.00	–	.994
	Father's education (Junior High vs. Postgrad)	2.20	0.21–23.09	.510
	Father's education (High school vs. Postgrad)	2.90	0.29–29.17	.367
	Father's education (Bachelor vs. Postgrad)	2.50	0.22–28.77	.462
	Urban origin (vs. Rural)	0.44	0.08–2.54	.360
	Suburban origin (vs. Rural)	0.49	0.04–5.71	.566
	Scholarship (No vs. Yes)	3.90	0.54–27.93	.176
	Family encouragement (No vs. Yes)	0.61	0.13–2.87	.533
	Relatives in pharmacy (No vs. Yes)	1.11	0.26–4.65	.888
**Pharmaceutical Industry**	Gender (Male vs. Female)	0.18	0.02–1.49	.111

Career Sector	Predictor	OR	95% CI	p-value
	Household income (Low vs. High)	~0.00	–	.994
	Household income (Middle vs. High)	~0.00	–	.994
	Father's education (Junior High vs. Postgrad)	1.83	0.24–14.06	.561
	Father's education (High school vs. Postgrad)	1.82	0.24–13.52	.561
	Father's education (Bachelor vs. Postgrad)	1.11	0.13–9.55	.923
	Urban origin (vs. Rural)	0.46	0.09–2.27	.339
	Suburban origin (vs. Rural)	0.41	0.04–3.96	.440
	Scholarship (No vs. Yes)	2.54	0.48–13.33	.271
	Family encouragement (No vs. Yes)	0.79	0.19–3.34	.753
	Relatives in pharmacy (No vs. Yes)	1.12	0.29–4.25	.872
**Academia**	Gender (Male vs. Female)	0.18	0.02–1.85	.149
	Household income (Low vs. High)	~0.00	–	.994
	Household income (Middle vs. High)	~0.00	–	.994
	Father's education (Junior High vs. Postgrad)	1.74	0.13–23.04	.675
	Father's education (High school vs. Postgrad)	2.28	0.18–28.66	.523
	Father's education (Bachelor vs. Postgrad)	1.28	0.08–20.08	.862
	Urban origin (vs. Rural)	0.28	0.04–2.16	.224
	Suburban origin (vs. Rural)	~0.00	–	.995
	Scholarship (No vs. Yes)	1.40	0.19–10.43	.746
	Family encouragement (No vs. Yes)	0.26	0.04–1.69	.158
	Relatives in pharmacy (No vs. Yes)	1.44	0.29–7.23	.657
**Government Regulation**	Gender (Male vs. Female)	0.33	0.03–3.32	.348
	Household income (Low vs. High)	1.20	0.24–6.06	.828
	Household income (Middle vs. High)	1.36	–	–

Career Sector	Predictor	OR	95% CI	p-value
	Father’s education (Junior High vs. Postgrad)	0.65	0.06–6.91	.722
	Father’s education (High school vs. Postgrad)	1.70	0.18–16.34	.648
	Father’s education (Bachelor vs. Postgrad)	0.66	0.05–8.02	.741
	Urban origin (vs. Rural)	0.44	0.07–2.78	.380
	Suburban origin (vs. Rural)	1.10	0.09–13.49	.939
	Scholarship (No vs. Yes)	1.53	0.23–10.33	.663
	Family encouragement (No vs. Yes)	0.66	0.13–3.37	.613
	Relatives in pharmacy (No vs. Yes)	3.69	0.69–19.79	.128

Note. Odds ratios (OR) less than 1 indicate lower odds relative to the reference group. Reference categories: Community Pharmacy (outcome), Female (gender), High income, Father postgraduate, Rural, Scholarship = Yes, Family encouragement = Yes, Relatives in pharmacy = Yes. Model fit: $\chi^2(66) = 57.6$, $p = .760$; Nagelkerke $R^2 = .122$.
